# shRNA-armed conditionally replicative adenoviruses: a promising approach for cancer therapy

**DOI:** 10.18632/oncotarget.8035

**Published:** 2016-03-10

**Authors:** Jie Zhang, Meng Ding, Kai Xu, Lijun Mao, Junian Zheng

**Affiliations:** ^1^ Jiangsu Key Laboratory of Biological Cancer Therapy, Xuzhou Medical College, Xuzhou, China; ^2^ Department of Urinary Surgery, The Affiliated Hospital of Xuzhou Medical College, Xuzhou, China

**Keywords:** conditionally replicating adenoviruses, RNA interference, cancer, target therapy

## Abstract

The small-interfering RNAs (siRNAs) have been employed to knockdown the expression of cancer-associated genes and shown some promise in cancer therapy. However, synthetic siRNA duplexes or plasmid mediated delivery of siRNAs have several problems, such as short half-life, low transfection efficiency and cytotoxicity associated with transfection. Conditionally replicating adenovirus (CRAds) as the delivery vector for short hairpin RNAs (shRNAs) could overcome these limitations and have shown augmented anti-tumor effects in experimental studies and preclinical trials. In this review, we summarize recent progress in the development of CRAds-shRNA for cancer treatment. Combination of CRAds-shRNA with chemotherapeutics, radiation, dendritic cells, monoclonal antibodies and small-molecule inhibitors will be necessary to eradicate cancer cells and cancer stem cells and achieve superior outcomes. The use of CRAd platform for efficient delivery of shRNAs and foreign genes will open a new avenue for cancer therapy.

## INTRODUCTION

**Figure 1:**
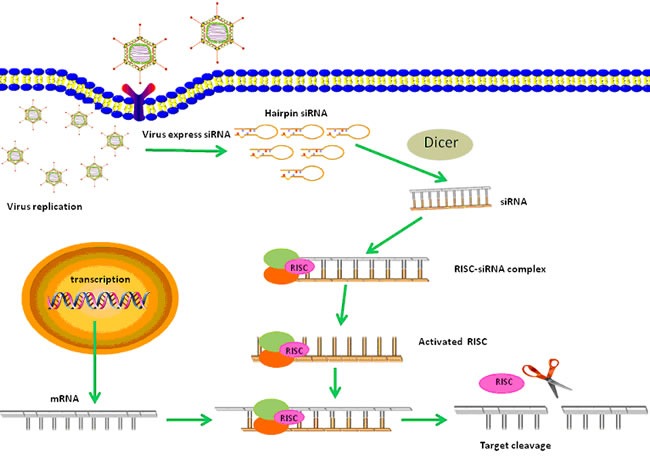
A schematic diagram of anti-tumor effects of CRAd-shRNA based therapy After CRAds infect and replicate in tumor cells, shRNAs are expressed within the nucleus where they spontaneously form hairpin RNAs and are transported to the cytoplasm. Then shRNAs are cleaved by Dicer into active siRNAs. Binding of the siRNA to RISC results in the activation of this complex, and after subsequent duplex unwinding, RISC facilitates the binding of target homologous mRNAs. Perfect binding sequences result in the cleavage and silencing of target genes.

RNA interference (RNAi) is a powerful tool for gene knockdown by sequence-specific post-transcriptional targeting [1]. Many studies have indicated the potential of RNAi for the treatment of cancer, especially those caused by abnormal gene expression. However, synthetic small-interfering RNA (siRNA) duplexes or plasmid mediated delivery of siRNAs bring many challenges, such as short half-life, low transfection efficiency and cytotoxicy associated with transfection [2]. In contrast, the application of RNAi technology in the context of conditionally replicative adenoviruses (CRAds) has various advantages, because CRAds replicate specifically in cancer cells and continuously knockdown the expression of oncogenes (Figure 1).

CRAds are natural or genetically modified viruses that selectively replicate in and kill neoplastic cells while sparing normal cells [3, 4]. Numerous CRAds have been developed in preclinical models and early clinical trials. Oncolytic Ad5-based viruses demonstrated efficacy and safety in preclinical and clinical trials [5–12]. Phase I human trials with CRAds have confirmed the overall safety of this approach. ONYX-015, an adenovirus, has been tested in randomized trials [13–16]. ONYX-015 was generated by deleting viral E1B 55KD gene, resulting in complete loss of E1B 55KD expression [17]. This modification enabled ONYX-015 to selectively infect and kill p53-deficient cancer cells without damaging normal cells, due to p53-mediated growth arrest [18]. Although ONYX-015 has been used extensively in clinical trials of cancer therapy, the clinical response rates have been suboptimal [19, 20]. One major obstacle is that ONYX-015 is only effective in p53-deficient tumors, and p53 is mutated in approximately 50% of all human cancers [21]. In addition, the deletion of E1B 55KD reduces the replication and production of Ads, resulting in reduced anti-tumor efficacy of CRAds.

To enhance the specificity and efficacy of CRAds for cancer treatment, oncolytic viruses have been modified to combine oncolytic replication with therapeutic transgenic expression. In this review we summarize recent progress in the utilization of CRAd-shRNA in cancer therapy and discuss novel strategies to develop highly efficient oncolytic Ads.

## STRATEGIES TO DEVELOP ONCOLYTIC ADENOVIRUS MEDIATED SHRNA

**Figure 2:**
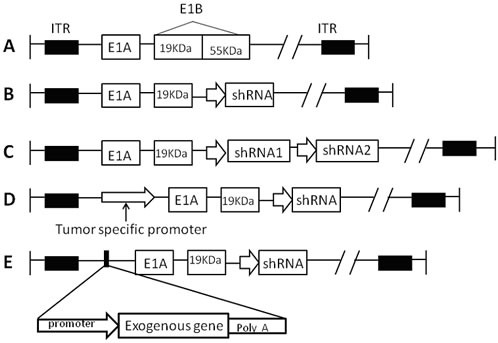
The schematic stucture of CRAd vector for shRNA **A.** Wild type adenovirus. **B.** In recombinant adenovirus, E1B55KD gene was replaced by one shRNA sequence expression cassette. **C.** CRAd vector harboring double-cistronic shRNA expression construct. **D.** CRAd armed with shRNA and tumor specific promoter. **E.** CRAd armed with shRNA and exogenous therapeutic genes.

Most adenoviral vectors used in RNAi delivery are derived from human serotypes 2 and 5. Carette et al. were the first to propose using shRNA with oncolytic viruses in cancer gene therapy [22]. This system can generate a two-pronged attack on tumor cells through oncogene knockdown and viral oncolysis. Up to now, the use of an oncolytic adenovirus platform for shRNA delivery can be divided into four forms (Figure 2).

### Oncolytic adenoviruses armed with one shRNA

The first strategy of oncolytic viruses armed RNAi involves the use of one shRNA. In the prototype, Carette et al. constructed CRAds encoding shRNAs against firefly luciferase and demonstrated that siRNAs expressed from CRAds could suppress the expression of firefly luciferase. In particular, the efficiency of silencing increased during viral replication [22]. Zhang et al. were the first to use oncolytic viral-transgene platform to achieve siRNA-mediated gene silencing that led to tumor cell death [23]. In recent years, our group has extensively tested this strategy using ZD55 which is defective in E1B gene. We have constructed ZD55-shRNAs against the genes overexpressed in cancer cells and examined the anti-cancer efficacy of recombinant ZD55-shRNAs such as ZD55-Ki67, ZD55-hTERT and ZD55-shMYCN [24–26]. We expanded this strategy to construct ZD55-SATB1, an oncolytic adenovirus carrying shRNA targeting SATB1, a oncogenic transcription factor [27]. We found that ZD55-SATB1 selectively replicated and significantly reduced SATB1 expression in DU145 and LNCaP cells. ZD55-SATB1 effectively inhibited the viability and invasion of DU145 and LNCaP cells *in vitro* and inhibited prostate cancer growth and metastasis in xenograft nude mice [27]. Our results indicate that the combination of oncolytic virotherapy and shRNA enhances the anti-tumor potency.

Yun et al. developed an E1A/E1B double mutant replicating adenovirus Ad-ΔE1Bmt7 [28], and constructed a series of Ad-ΔE1Bmt7 armed with shRNAs targeting the key mediators in angiogenesis, the armed Ad-ΔE1Bmt7 demonstrated greater anti-tumor and anti-angiogenesis efficacy than Ad-ΔE1Bmt7 alone, when injected intratumorally in subcutaneous models of glioma, hepatocellular carcinoma and lung carcinoma [29–31]. Recently, Chen and colleagues employed adenovirus-mediated siRNA to knockdown FAT10 expression in hepatocellular carcinoma cells. These replicating viruses specifically silenced target gene and potently inhibited tumor growth *in vivo* [32].

### Oncolytic adenoviruses armed with dual or multiple shRNAs

The second strategy involves the use of dual or multiple shRNAs in one Ad vector. Jazag et al. were the first to establish the method for simultaneous silencing of multiple targets by shRNA-expressing RNAi plasmid vectors [33]. The multi-siRNA engineering technology has been applied in the construction of adenoviral vectors [34]. Motegi et al. developed an efficient Ad vector-mediated RNAi system carrying four shRNA-expression cassettes [35]. Ad-multi-shRNA vectors showed enhanced gene silencing compared to conventional Ad vectors containing a single shRNA-expression cassette. Our team tested a ‘dual target’ approach by constructing double siRNAs (targeting Ki67 and hTERT) delivered by one oncolytic adenovirus [36]. The double siRNAs armed oncolytic adenoviruses could kill renal cancer cells effectively. Another recent study reported that single promoter-driven multi-cistronic shRNAs for XIAP, Akt and Bcl-2 effectively silenced multiple target genes [37].

We postulate that simultaneous targeting of two or multiple tumor-specific genes using oncolytic adenovirus and siRNA enhances therapeutic efficacy against tumors, but could augment the inherent risks, including immune responses, cytotoxicity, off-targeting and oversaturation of endogenous pathways. Moreover, the optimization of siRNA is needed for stable knockdown of multiple genes. These factors should be taken into account when combinatorial RNAi is selected for gene therapy.

**Table 1:** Typical examples of CRAds armed with shRNA

Function of targets	Cancer types	Effects	Genes targeted	References
Regulation of cell cycle	Renal carcinoma	Antitumor	Ki67	[27]
proto-oncogenes	Neuroblastoma	Antitumor	MYCN	[29]
Transcription factor	Prostate cancer	Antitumor	SATB1	[30]
Pro-angiogenic factor	Glioma, hepatocellular carcinoma (HCC)	Antitumor	VEGF	[32–34]
Ubiquitin-related family	HCC	Antitumor	FAT10	[35]
tumor growth and metastasis	HCC	Antitumor	FAK	[58]
Cell immortalization and tumorigenesis	Cervical cancer, renal carcinoma, fibrosarcoma	Antitumor	hTERT	[28],[41]
tumor growth, invasion, and metastasis	prostate cancer, gastric carcinoma, breast cancer	Antitumor	EphA3	[56]
Inhibitor of apoptotic protein	Colon cancer, renal carcinoma	Silence target gene	XIAP, Bcl-2, Akt	[42],[45]
	Cervical cancer, adenocarcinoma, fibrosarcoma	Enhance chemotherapy efficacy	Apollon	[76]
	Colorectal cancer	Enhance chemotherapy efficacy	Survivin	[72]
DNA double strand break repair	Colon cancer	Enhance radiotherapy efficacy	DNA-PKCs	[82]

### Oncolytic adenoviruses armed with shRNA and therapeutic genes

Recently, cancer gene therapy has focused on utilizing cytokines, tumor suppressor, and apoptosis related genes as therapeutic genes [38, 39]. CRAds armed with a variety of transgenes enhance the killing of cancer cells, modulate tumor microenvironment, and stimulate immune response to the tumor. Replicating oncolytic Ads are able to infect and deliver therapeutic genes to adjacent cells in addition to those infected initially. Furthermore, CRAds induce high expression of therapeutic genes through increased adenoviral replication, and restrict the expression of therapeutic genes to cancer cells. Central to this strategy, however, is the engineering of Ads vectors that can efficiently deliver therapeutic genes of interest and siRNAs targeting oncogenes together to solid tumors. Pan et al. utilized ZD55 to deliver X-linked inhibitor-of-apoptosis protein (XIAP) shRNA and TRAIL into hepatocellular carcinoma cells and demonstrated significant reduction of XIAP expression and potent anti-tumor activity both in hepatocellular carcinoma cells and in tumor animal model. However, the effect *in vivo* was not as adequate as that of *in vitro* experiments, particularly at late stage [40]. The phenomena may suggest that conventional oncolytic adenoviral vectors are not so efficient for long-term siRNA delivery, probably due to the elimination of oncolytic adenovirus by host or the expression of noncoding adenovirus VA RNAI and VA RNAII that have the capacity to suppress RNAi at late stage of infection [41]. Therefore, these oncolytic adenoviruses need to be further modified, such as deleting VA RNAI and VA RNAII, to provide a more efficient oncolytic vehicle for siRNA delivery in cancer therapy.

### Oncolytic adenoviruses armed with shRNA and tumor specific promoter

The nonspecific native tropism of the adenovirus limits the efforts to target cancer cells specifically, and increases the potential for side effects. One strategy is the use of tumor-specific promoters (TSPs) to selectively drive viral E1 expression to increase tumor specificity. This is typically accomplished by placing viral E1A gene under the control of an exogenous promoter that is active or induced in the particular cancer that is being targeted. The most commonly used promoter that augments virus specificity is hTERT, which is active in 85-90% of tumor tissues and is detectable in the early stages of malignancy [42]. hTERT promoter is inactive in most normal host tissues but displays high activity in a majority of human cancers [43, 44]. Telomerase is thus considered as an ideal tumor-specific regulator of oncolytic adenoviruses [45]. Zhao et al. tested this approach by combining Ad-TERTp with siRNA targeting EphA3, a potential oncogene. They found that Ad-TERTp-E1A-EphA3 shRNA had 3.5- and 1,400- fold greater ability to kill EphA3 and TERT expressing tumor cells compared to Ad-TERTp-E1A-NC and Ad-ΔE1A-EphA3 shRNA, respectively, while had little effect on cells that modestly expressed EphA3 and TERT. The anti-tumor efficacy of Ad-TERTp-E1A- EphA3 shRNA was further validated *in vivo* [46].

We constructed oncolytic virus G250-Ki67, and G250 promoter-derived CRAds carrying Ki67-siRNA could efficiently amplify and deliver Ki67-siRNA in renal cancer cells, leading to inhibited proliferation and enhanced apoptosis [47]. A recent study reported the engineering of adenovirus Ad-hTERT-HREAF (named SG505). shRNA against focal adhesion kinase (FAK) was inserted into SG505 to form Ad-hTERT-HREAF-shRNA (called SG505-siFAK). Both replicative and replication-defective adenoviruses carrying FAK-shRNA significantly inhibited FAK expression and efficiently suppressed the growth of liver cancer cells with minor effect to normal cells [48].

## CRADS BASED COMBINATION THERAPY

The monotherapy of adenovirus has demonstrated limited efficacy in a clinical setting [49]. Comprehensive therapy has become dominant feature in improving tumor therapy [50–52]. Therefore, oncolytic adenoviruses have been used in combination with other tumor treatment strategies, including gene therapy, cell therapy, and traditional radiation and chemotherapy [53–56].

### CRAds in combination with chemotherapy

Preclinical and clinical trials support that CRAds gene therapy and chemotherapy have complementary or synergistic effects, leading to better anti-tumor effect than either treatment alone. One effective approach in the treatment of malignant tumors is direct injection of CRAds combined with chemotherapeutic agents. Another means of combined therapy is the use of CRAd-shRNA to knockdown genes associated with chemoresistance in tumor cells.

Apollon is a membrane-associated inhibitor of apoptosis and is upregulated in chemoresistant cancer cells [57]. Chu et al. constructed ZD55-siApollon to knockdown Apollon and showed that ZD55-siApollon inhibited tumor progression *in vivo*, but the tumors were not eliminated. In contrast, complete tumor eradication was observed when tumors were co-injected with ZD55-siApollon and 5-FU [58]. Another recombinant adenovirus armed with shRNA targeting surviving was administrated together with 5-FU and inhibited cancer metastasis both *in vitro* and *in vivo* [59].

### CRAds in combination with radiotherapy

Total radiation dose that can be delivered to cancer is limited by the damage to normal tissues that are irradiated during radiotherapy [60]. Recently, several studies have shown that the combination of radiotherapy and oncolytic adenovirus mediated gene therapy has synergistic suppressive effects on the growth of various cancer cells [61–63]. Notably, a prospective randomized phase 2 trial showed that combining oncolytic adenovirus mediated cytotoxic gene therapy with radiation therapy did not exacerbate the most common side effects of prostate radiation therapy [64].

Takashi et al. engineered a replication-deficient adenovirus encoding shRNA targeting DNA-PKcs to treat human HCT116 colon cancer cells, leading to increased radiation sensitivity [65]. When CRAds targeted to telomerase-positive tumor cells was used in conjunction with non-replicative adenovirus harboring DNA-PKcs-shRNA, the efficiency of tumor-specific knockdown of DNA-PKcs was enhanced significantly, contributing to significant anti-tumor efficacy of concurrent radiation therapy. These results suggest that shRNA mediated DNA-PKcs knockdown in combination with replicative adenovirus is a promising approach for the sensitization of solid tumors to radiation therapy.

### CRAds in combination with immunotherapy

For cancer immunotherapy, different kinds of immune cells have been used including dendritic cells (DCs), cytokine-induced killer (CIK) cells, cytotoxic T lymphocytes (CTLs) and natural killer (NK) cells [66–70]. DCs are highly efficient and specialized antigen-presenting cells. However, tumor tissues produce immunosuppressive molecules such as vascular endothelial growth factor (VEGF) and transforming growth factor β to induce an immunosuppressive microenvironment and inhibit the function of tumor-associated DCs [71]. To augment the anti-tumor efficiency of cytokine-mediated immunotherapy, Huang et al. administered IL-12 and 4-1BBL co-expressing oncolytic adenovirus with DCs [72]. The combination of CRAds and DCs elicited greater anti-tumor and anti-metastatic effects than either treatment alone. The enhanced anti-tumor immune response seems to be mediated by enhanced cytolytic activity of CTLs and IFN-γ releasing immune cells [72].

pRb/E2F pathway is abnormal in many solid tumors [73]. Yang et al. used an E2F-1 targeted oncolytic adenovirus in combination with CIK cells for colorectal cancer therapy, and observed strong anti-tumor effect [74]. Later, they inserted IL-15 gene into the E3 region of the adenovirus and found that human IL-15 expressing oncolytic adenovirus (Ad-E2F/IL15) showed stronger anti-tumor effect than simple oncolytic viruses (Ad-E2F). In addition, the therapeutic effect of Ad-E2F/IL15 in combination with CTLs was clearly stronger than that of single application of oncolytic viruses or CTL [75]. In conclusion, the combination of CRAds and adoptive immune cells can achieve synergistic efficacy for tumor therapy.

## CRADS OVERCOME THE CHALLENGES OF CURRENT CANCER THERAPY

Despite the advantages of CRAds for cancer therapy, two important issues remain as the big challenges. First, cancer stem cells (CSCs) are thought to account for tumor relapses. Second, clinical trials using advanced anti-cancer therapies fail to reach the outcomes of preclinical studies due to limited distribution throughout solid tumors and quick degradation of the therapeutic payload [76]. Therefore, it is worthwhile to optimize the strategy of siRNA armed CRAds to improve cancer therapy.

### CRAds for killing cancer stem cells

Pierce speculated that tumors contain a small number of malignant stem cells that maintain stem cell function and give rise to progenitor cells with various degree of differentiation [77]. Currently, CSCs have been isolated from the brain, pancreas, ovarian, colorectal, lung, head and neck, breast, liver and a variety of pediatric cancers [78–82]. The stem cell-like properties of CSCs confer resistance to traditional chemotherapy and radiotherapy [82].

A number of studies have explored the use of oncolytic Ads for CSCs targeting. Ad5/3-Δ24 and Ad5.pK7-Δ24 had a 24-base pair deletion in the viral E1A gene, which disrupted the Rb-binding capacity of E1A protein and thereby conferred conditional replication only in cells deficient in Rb/p16 pathway [83, 84]. The RGD peptide allowed the virus to bind and enter the cell through cell surface integrins αvβ3/5, and effectively kill breast cancer cells [85]. Eriksson et al. evaluated the oncolytic potency of CRAds for CD44^+^CD24^low/−^ breast cancer CSCs *in vitro* and *in vivo* [86]. When CD44^+^CD24^low/−^ cells were injected into the fat pads of SCID mice, tumors formed rapidly, but CD44^+^/CD24^−/low^ cells infected with Ad5/3-Δ24 showed slower tumor formation [86].

Oncolytic adenovirus Delta24-RGD represents a promising therapeutic agent for malignant glioma. Delta24-RGD showed efficacy against glioma *in vitro* and *in vivo* [87], and phase I clinical trial has been recently completed [88]. Histone deacetylase inhibitors (HDACi) are novel anti-cancer drugs and the combination of HDACi and Delta24-RGD showed synergistic anti-tumor activity in patient-derived glioblastoma stem-like cells (GSCs). Meanwhile, the limited toxicity of HDACi to normal human astrocytes makes these drugs interesting candidates for combination with Delta24-RGD for cancer therapy [89].

Moreover, transcriptional targeting mediated by adenoviral vectors using promoters active in CSCs may be a powerful approach for the eradication of CSCs [90]. With the progress made in CSCs biology, next-generation viruses can be developed that target specific CSCs antigens and signaling pathways that promote tumorigenesis.

### MSCs mediated systemic delivery of CRAds

The systemic administration of oncolytic virus is often inefficient, because the virus is susceptible to neutralization by blood components, especially antibodies, and sequestration to healthy organs [91]. An alternative strategy for *in vivo* delivery of oncolytic viruses is using cell carriers that chaperone viral delivery to the tumor nodules. Carrier cells for oncolytic virus delivery could shield the virus from host defenses and direct them toward tumors. Mesenchymal stromal cells (MSCs) are interesting candidates as carrier cells, because they are easily obtained, cultivated and propagated *in vitro*, and can home to tumor microenvironments [92]. Using MSCs as a delivery system could improve the distribution of CRAds into tumor, minimize the off-target toxicity and enhance therapy efficacy.

MSCs have been employed to deliver oncolytic virus which subsequently infect and replicate within malignant cells and eradicate the tumors [93–97]. When Ad5-Delta24-RGD loaded into MSCs was administered to mice with intracranial glioma, there was 80% increase in median survival (42 days in controls compared to 75.5 days in treated animals) [97]. Delivery of CRAd by human MSCs was 46-fold more efficient than the injection of CRAd alone, indicating that human MSCs migrate and deliver CRAd to distant glioma cells [95]. It was demonstrated that engineering post-entry steps of oncolytic virus replication could dramatically increase virus dose released by MSCs [98]. The Ad5/3 chimeric OAd capsid increased the entry into human bone marrow-derived MSCs and primary pancreatic cancer cells. The combination of MSCs mediated virus delivery with 5-FC/5-FU prodrug activation showed very strong tumor cell killing ability [98].

## CONCLUDING REMARKS

CRAds can be used as gene delivery vehicles to tumors, in addition they independently induce oncolysis and avoid the damage of adjacent normal cells. A variety of strategies have been designed to enhance the oncolytic effects of CRAds. Among them, two major strategies of CRAds armed with shRNA have been pursued: i) improve the efficiency and selectivity by silencing multiple target genes, co-expression of tumor suppressor genes or using tumor specific promoters; ii) improve CRAds delivery and reduce virus clearance by using MSCs as cell delivery vehicles. Combination therapy with chemotherapeutics, radiation, dendritic cells, monoclonal antibodies, small-molecule inhibitors will be necessary to eliminate cancer cells and CSCs to achieve superior outcomes. The use of CRAd platform for shRNA and transgenic delivery will open a new avenue for cancer therapy.
